# Reviews

**Published:** 1862-04

**Authors:** 


### REVIEWS.

*Border Lines of Knowledge in some provinces of Medical Science; an introductory Lecture delivered before the Medical Class of Harvard University, Nov. 6th,* 1861, *by* Oliver Wendell Holmes, M. D., *Parkman Professor of Anatomy and Physiology. Boston:* Ticknor & Fields, 1862.

In noticing this Lecture we desire to make a few brief extracts from its pages, which are full of thought and interest and poetry. All who know Dr. Holmes—and who does not—will understand that it is original, suggestive, truthful, progressive, instructive, sublime beyond description, and above all, full of the poetry of "high and noble thought."

"Science is the topography of ignorance. From a few elevated points we triangulate vast spaces, enclosing infinite unknown details. We cast the lead, and draw up a little sand from abysses we shall never reach with our dredges.

The best part of our knowledge is that which teaches us where knowledge leaves off and ignorance begins. Nothing more clearly separates a vulgar from a superior mind, than the confusion in the first between the little that it truly knows, on the one hand, and what it half knows and what it thinks it knows, on the other."

"Chemistry includes the art of separating and combining the elements of matter, and the study of the changes produced by these operations. We can hardly say too much of what it has contributed to our knowledge of the universe and our power of dealing with its materials. It has given us a *catalogue raisonne* of the substances found upon our planet, and shown how everything living and dead is put together from them. It is accomplishing wonders before us every day, such as Arabian story-tellers used to string together in their fables. It spreads the sensitive film on the artificial retina which looks upon us through the optician's lens for a few seconds, and fixes an image that will outlive its original. It questions the light of the sun, and detects the vaporized metals floating around thegreat luminary—iron, sodium, lithium, and the rest—as if the chemist of our remote planet could fill his bell-glasses from its fiery atmosphere.* It lends the power which flashes our messages in thrills that leave the lazy chariot of day behind them. It seals up a few dark grains in iron vases, and lo! at the touch of a single spark, rises in smoke and flame a mighty Afrit with a voice like thunder and an arm that shatters like an earthquake. The dreams of Oriental fancy have become the sober facts of our every-day life, and the chemist is the magician to whom we owe them.

To return to the colder scientific aspect of chemistry. It has shown us how bodies stand affected to each other through an almost boundless range of combinations. It has given us a most ingenious theory to account for certain fixed relations in these combinations. It has successsully eliminated a great number of proximate compounds, more or less stable, from organic structures. It has *invented* others which form the basis of long series of well-known composite substances. In fact, we are perhaps becoming overburdened with our list of proximate principles, demonstrated and hypothetical."

"Descriptive anatomy, as known from an early date, is to the body what geography is to the planet. Now geography was pretty well known so long ago as when Arrowsmith, who was born in 1750, published his admirable maps. But in that same year was born Werner, who taught a new way of studying the earth, since become familiar to us all under the name of *Geology*.

What geology has done for our knowledge of the earth, has been done for our knowledge of the body by that method of study to which is given the name of *General Anatomy*. It studies, not the organs as such, but the elements out of which the organs are constructed. It is the geology of the body, as that is the general anatomy of the earth. The extraordinary genius of Bichat, to whom more than any other we owe this new method of study, does not require Mr. Buckle's testimony to impress the practitioner with the importance of its achievements. I have heard a very wise physician question whether any important result had accrued to practical medicine from Harvey's discovery of the circulation. But Anatomy, Physiology and Pathology, have received a new light from this novel method of contemplating the living structures, which has had a vast influence in enabling the practitioner at least to distinguish and predict the course of disease. We know as well what differences to expect in the habits of a mucous and of a serous membrane, as what mineral substances

* Scientific Annual for 1861.—FAIRBAIRN'S Address before the British Association, 1861.

to look for in the chalk or the coal measures. You have only to read Cullen's description of inflammation of the lungs or of the bowels, and compare it with such as you may find in Laennec or Watson, to see the immense gain which diagnosis and prognosis have derived from general anatomy."

"But I am very willing to confess a great jealousy of many agents, and I could almost wish to see the Materia Medica so classed as to call suspicion upon certain ones among them.

Thus the *alien elements*, those which do not properly enter into the composition of any living tissue, are the most to be suspected—mercury, lead, antimony, silver, and the rest, for the reasons I have before mentioned. Even iodine, which, as it is found in certain plants, seems less remote from the animal tissues, gives unequivocal proofs from time to time that it is hostile to some portions of the glandular system.

There is, of course, less *prima facie* objection to those agents which consist of assimilable elements, such as are found making a part of healthy tissues. These are divisible into three classes—foods, poisons, and inert, mostly because insoluble, substances. The food of one animal or of one human being is sometimes poison to another, and *vice versa;* inert substances may act mechanically, so as to produce the effect of poisons; but this division holds exactly enough for our purpose.

Strictly speaking, every poison consisting of assimilable elements may be considered as *unwholesome food.* It is rejected by the stomach, or it produces diarrhœa, or it causes vertigo or disturbance of the heart's action, or some other symptom for which the subject of it would consult the physician, if it came on from any other cause than taking it under the name of medicine. Yet portions of this unwholesome food which we call medicine, we have reason to believe, are assimilated; thus, castor-oil appears to be partially digested by infants, so that they require large doses to affect them medicinally. Even that deadliest of poisons, hydrocyanic acid, is probably assimilated, and helps to make living tissue, if it do not kill the patient, for the assimilable elements which it contains, given in the separate forms of *amygdalin* and *emulsin,* produce no disturbance, unless, as in Bernard's experiments, thay are suffered to meet in the digestive organs. A medicine consisting of assimilable substances being then simply *unwholesome food,* we understand what is meant by those *cumulative* effects of such remedies often observed, as in the case of digitalis and strychnia. They are precisely similar to the cumulative effects of a salt diet in producing scurvy, or of spurred rye in producing dry gangrene. As the effects of such substances are a violence to the organs, we should exercise the same caution with regard to their use that we would exercise about any other kind of poisonous food—partridges at certain seasons, for instance. Even where these poisonous kinds of food seem to be useful, we should still regard them with great jealousy. *Digitalis* lowers the pulse in febrile conditions. *Veratrum viride* does the same thing. How do we know that a rapid pulse is not a normal adjustment of nature to the condition it accompanies? Digitalis has gone out of favor; how sure are we that *Veratrum viride* will not be found to do more harm than good in a case of internal inflammation, taking the whole course of the disease into consideration? Think of the change of opinion with regard to the use of opium in *delir-*

*ium tremens,* (which you remember is sometimes called *dilirium vigilans,*) where it seemed so obviously indicated, since the publication of Dr. Ware's admirable essay. I respect the evidence of my contemporaries, but I can not forget the sayings of the Father of medicine—*Ars longa, judicium difficile.*

I am not presuming to express an opinion concerning *Veratrum viride,* which was little heard of when I was still practicing medicine. I am only appealing to that higher court of experience which sits in judgment on all decisions of the lower medical tribunals, and which requires more than one generation for its final verdict.

Once change the habit of mind so long prevalent among practitioners of medicine; once let it be everywhere understood that the presumption is in favor of food, and not of alien substances, of innocuous, and not of unwholesome food, for the sick; that this presumption requires very strong evidence in each particular case to overcome it; but that, when such evidence is afforded, the alien substance or the unwholesome food should be given boldly, in sufficient quantities, in the same spirit as that which the surgeon lifts his knife against a patient—that is, with the same reluctance and the same determination—and I think we shall have and hear much less of charlatanism in and out of the profession. The disgrace of medicine has been that colossal system of self-deception, in obedience to which mines have been emptied of their cankering minerals, the vegetable kingdom robbed of all its noxious growths, the entrails of animals taxed for their impurities, the poison-bags of reptiles drained of their venom, and all the inconceivable abominations thus obtained thrust down the throats of human beings suffering from some fault of organization, nourishment, or vital stimulation.

Much as we have gained, we have not yet thoroughly shaken off the notion that poison is the natural food of disease, as wholesome aliment is the support of health. Cowper's lines, in The Task, show the matter-of-course practice of his time:

> "He does not scorn it, who has long endured

> A fever's agonies, and *fed on drugs.*"

---

*Clinical Lectures on the Diseases of Women and Children, by* GUNNING S. BEDFORD, A. M., M. D, *Professor of Obstetrics, the Diseases of Women and Children, and Clinical Obstetrics, in the University of New York:* WILLIAM WOOD, 1862.

The value of clinical observation and instruction is rapidly receiving a truer estimate by the profession, and its importance is so fully appreciated by the best and most experienced teachers, that every effort is made to afford students of medicine the greatest possible opportunity. Prof. Bedford established in 1850, in connection with the University of New York an *Obstetric Clinic,* for the purpose of increasing the facilities for the practical study of the diseases of women. There have been presented to his classes, over eight thousand cases of disease, and this volume contains a summary, as it were, of the various maladies peculiar to women and children which have been discussed and treated in this clinic. The practicing physician has thus offered him a most invaluable guide to the correct pathology and successful treatment of the diseases of females, and it is remarkable with what distinctness these hitherto obscure diseases are presented by the colloquial style of the clinical lecture. Many topics which one would most desire to have explained, are easily, naturally and scientifically discussed, in a manner much more satisfactory than could possibly be expected in a systematic treatise, strictly speaking, upon the pathology and treatment of uterine diseases. The patient is brought before you; you make *all the inquiries* you desire, and hear the reply, when the diagnosis, pathology, and treatment are fully given, and many incidental suggestions made, so that we more truly receive the advantages of actual practice than has ever before been attained in reading a book.

The comparatively new field of uterine pathology has been as fully explored and mapped as accurately and scientifically as the present advanced state of medical science will permit, while the great value of this volume consists in the variety of instruction it contains, the graphic style in which it is presented, and the truthful, practical character of the doctrines advanced. The author receives the very rare and unusual compliment of having this book translated into the *French and German languages*, and we most heartily congratulate him upon this exceedingly rare tribute to American Medical Authorship. While this translation is the highest compliment Prof. Bedford could possibly receive, and shows conclusively the estimate which is properly placed upon his labors abroad, he is also receiving the highest testimonials which can be presented, that his efforts are also appreciated by his own countrymen, since the productions of no American medical author has ever been better received than the two last works of Prof. Bedford; *Principles and Practice of Obstetrics* and *Diseases of Women and Children;* and no two books ever deserved a more hearty welcome. We sincerely hope that every American physician will avail himself of these volumes.

---

*Commentaries on the Surgery of the War in Portugal, Spain, France, and the Netherlands, from the battle of Rolica in* 1808, *to that of Waterloo, in* 1815; *with additions relating to those in the Crimea in* 1854, 1855, *showing the improvements made during and since that period in the great art and science of Surgery on all the subjects to which they relate. Revised to October,* 1855, *by* G. J. GUTHRIE, F. R. S.

*Sixth edition. Philadelphia:* J. B. LIPPINCOTT & Co., 1862. *For sale in Buffalo by* BREED, BUTLER & Co.—$1.50.

This volume consists of thirty Lectures upon the Surgery of War, which have been published in the *Lancet,* at intervals, and which now with an addenda, compose this work. The precepts laid down are the result of the experience acquired in the War of the Peninsular, from the first battle of Rolica in 1808 to the last in Belgium, of Waterloo in 1815, "which altered, nay overturned, nearly all those which existed previously to that period, on all points to which they relate; points as essential in the surgery of domestic as well as military life."

We have read these lectures with great interest, and find that the whole field of Military Surgery has been fully covered. In saying this we shall be understood as meaning that general Surgery is fully taught and explained. The various operations are described as performed by some of the best surgeons or by *all* good operators. The advantages and objections one mode has over another plan of operation, is very fully discussed, and the various improvements made in surgery during the last fifty years explained. At the present time this book must be found of the highest value in determining points yet remaining uncertain, since the experience of such an immense field has been made to contribute to this end.

Physicians of the Army, or those about to enter this service, should be in possession of the teaching of these lectures. They will be found entertaining, instructive and highly valuable, contributing to the general intelligence as well as the special ability of those who carefully peruse them.

---

*American Journal of Ophthalmology—Edited by* JULIUS HOMBERGER, *M. D., Pupil of Prof. De Graefe; late Assistant to Dr. Sichel, Paris; Member of the Societe Universelle d' Ophthalmologie, etc., etc.*

The *American Journal of Ophthalmology* is, as its name implies, exclusively devoted to Ophthalmic Medicine and Surgery.

The Editor does not deem it necessary to justify the new enterprise in answer to the prejudiced reproach, that the Medical Profession is opposed to specialists. This opposition is an undeniable fact, probably arising from the number of quacks, assuming the title of Oculists. But what would be thought of a general who would give up his study of military science, and his title, because, forsooth, a number of Falstaffs have donned his honors? Yet this would be just as absurd and unjustifiable, as to abandon the field of specialism on account of impostors, who have usurped it. Besides, there certainly are many medical men, who have long felt the necessity of an organ of Ophthalmology, more especially portraying the advances of this branch, than the general medical journals are enabled to do.

The Editor's connection with the most celebrated European Oculists will give an additional value and character to the Journal; reports of operations, original articles and periscopes, from American as well as foreign sources, will make it national as to the American profession and cosmopolitan as to science.

Each number will contain a department for "Answers to Correspondents," and there the Editor will endeavor to save, as far as possible, the advice of a Consulting Oculist.

Contributions in accordance with the present state of physiological and anatomical research, and therapeutical and operative procedure, are earnestly solicited, and if used, will be liberally paid for.

The Journal will be published bi-monthly, at $2 per annum, payable on receipt of the first number.

All wishing to subscribe are requested to send their names to BAILLIERE BROTHERS, 440 Broadway.

Inquiries, Communications, Exchanges, etc., etc., must be forwarded to DR. HOMBERGER, Editor American Journal of Ophthalmology, 61 Ninth Street, New York.

